# Quantitative Analysis of Topographic Crosstalk in DART‐ESM Arising from Feedback‐Loop‐Delay‐Induced Contact Stiffness Variations in Battery Materials

**DOI:** 10.1002/smtd.70763

**Published:** 2026-06-11

**Authors:** Dongyan Chen, Junki Lee, Chaeeun Song, Seung Hee Han, Youngwoo Choi, Chaewon Gong, Aditi Saha, Nam‐Soon Choi, Jong Min Yuk, Seungbum Hong

**Affiliations:** ^1^ Department of Materials Science and Engineering Korea Advanced Institute of Science and Technology (KAIST) Daejeon South Korea; ^2^ Department of Chemical and Biomolecular Engineering Korea Advanced Institute of Science and Technology (KAIST) Daejeon South Korea; ^3^ KAIST Institute for Climate Energy, and Environment Korea Advanced Institute of Science and Technology (KAIST) Daejeon Republic of Korea

**Keywords:** electrochemical strain microscopy, ionic transport, nanoscale electrochemistry, resonance tracking, solid electrolyte

## Abstract

Electrochemical Strain Microscopy (ESM) is widely used to probe nanoscale ion dynamics in battery materials, particularly at grain boundaries, where ionic transport is often proposed to be localized. However, interpretation of ESM signals remains challenging because topography‐induced artifacts can artificially enhance the measured response. Here, topographic crosstalk arising from contact stiffness variation induced by feedback‐loop delay is quantitatively analyzed in Dual AC Resonance Tracking (DART)‐ESM using ionically inactive single‐crystal silicon as a reference material. Artificial trench structures are introduced to emulate grain‐boundary‐like topography commonly encountered in battery electrodes and solid electrolytes. Simple harmonic oscillator (SHO) analysis of contact‐resonance dynamics shows that ESM amplitude enhancement can arise from contact stiffness variations independent of ionic motion. These silicon‐based measurements provide a practical reference for identifying topographic crosstalk and estimating its magnitude. Reproducibility is confirmed across multiple silicon calibration samples and further validated in practical battery materials, including a graphite anode and a Na_2_Zn_2_TeO_6_ (NZTO) solid electrolyte, indicating that such artifacts are inherent to DART‐ESM under practical measurement conditions. Cooling Cross‐Section Polishing (CCP) effectively suppresses these artifacts by reducing surface roughness and stabilizing contact resonance. These results provide a practical framework for reliable interpretation of nanoscale electrochemical activity in battery materials.

## Graphical abstract

Interpretation of Dual AC Resonance‐Tracking Electrochemical Strain Microscopy (DART‐ESM) signals is complicated by topography‐induced artifacts that distort measured responses. Using silicon with artificial trench structures as a reference, this study quantifies artifact magnitude and demonstrates that Cooling Cross‐Section Polishing (CCP) effectively suppresses these artifacts, enabling more reliable evaluation of nanoscale electrochemical activity and ionic transport in battery materials.

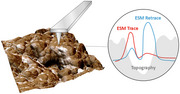

## 1 Introduction

The ability to accurately map ion dynamics at the nanoscale is particularly important as researchers strive to develop next‐generation practical batteries with improved capacity, charging rates, and cycle life [1, 2, 3, 4, 5]. Electrochemical Strain Microscopy (ESM) has emerged as a powerful scanning probe technique for investigating the nanoscale electrochemical processes in energy storage materials, particularly for lithium ion batteries [6, 7, 8, 9, 10]. ESM shares the same experimental setup as Piezoresponse Force Microscopy (PFM), but the two techniques differ in their contrast mechanisms. While PFM probes piezoelectric responses [11, 12], ESM detects the picometer scale mechanical deformation (electrochemical strain) that occur during ion insertion and extraction processes [13, 14, 15, 16], providing critical insights into local ionic transport, reactivity, and degradation mechanisms [17, 18].

However, the interpretation of ESM data is often complicated by artifacts that can mask or distort the true electrochemical response signals [19, 20, 21]. Since its development in the early 2010s, ESM has been applied to various battery materials, and increasing attention has been paid to the challenge of separating intrinsic electrochemical responses from measurement artifacts [22, 23, 24, 25]. Early work by Balke et al. established the fundamentals of the ESM technique and demonstrated its application to lithium‐ion battery cathodes while acknowledging the potential for topographic crosstalk in measurements [26].

The measured ESM response represents a convolution of electrochemical activity with multiple coupled mechanical and instrumental factors [19, 22, 23, 24, 25, 27, 28, 29, 30, 31, 32]. These factors can be broadly classified into excitation‐related effects, which govern how the cantilever is driven, and detection‐related effects, which determine how the cantilever response is modified and measured. The former includes the driving force, such as electrostatic or electromechanical excitation, as well as the driving frequency. The latter include resonance frequency, quality factor (Q factor), response phase, and other dynamic properties of the cantilever–sample system.

In resonance‐enhanced ESM and PFM measurements, it is well recognized that spatial variations in the contact resonance frequency can induce pronounced changes in the detected amplitude signal [33]. To mitigate topographic crosstalk, several methodological advances have been proposed. Rodriguez et al. made a critical advancement with the Dual AC Resonance‐Tracking (DART) method, which tracks contact resonance frequency, amplifies the inherently weak ESM signals, and substantially reduces topographic crosstalk that previously confounded data interpretation [20, 34]. Jesse et al. introduced band excitation techniques and multivariate statistical methods to help separate true electrochemical responses from topography‐related signals [35, 36, 37, 38]. While these studies have provided important mechanistic insights and methodological improvements, topographic crosstalk still cannot be completely eliminated.

Among the many factors that complicate interpretation of ESM signals, contact stiffness plays a particularly important role because it serves as an intermediate parameter connecting the local tip–sample interaction with the cantilever's dynamic response. While contact stiffness itself can vary with changes in contact area, local elastic properties, and instrumental effects such as feedback delay, these diverse influences are effectively reflected through a single measurable parameter [19, 23, 25, 28, 29]. In this sense, contact stiffness provides a unified description of multiple contributions. By contrast, many other contributing factors depend more directly on instrument‐specific parameters, such as cantilever stiffness, as well as on excitation conditions, including the applied AC voltage or DC bias, making their effects less universal and more difficult to generalize within a unified framework.

Because complete isolation of all contributions remains highly challenging, this work focuses on one of the most experimentally accessible and unavoidable pathways: AFM feedback‐induced variations in loading force and their role in generating topographic crosstalk through changes in contact stiffness. To investigate this effect, an ionically inactive single‐crystal silicon wafer is employed as a model system, enabling the separation and quantitative analysis of topographic crosstalk arising from contact stiffness variations under DART‐mode ESM. Artificial trenches are introduced to mimic grain‐boundary‐like features commonly observed in battery materials. By comparing experimentally measured local variations in contact resonance frequency with those predicted by a simple harmonic oscillator (SHO) model, the influence of tip deflection (loading force) on the resonance frequency is first examined at the trenches. This influence is then evaluated across the full scan via cross‐correlation analysis of the line profile, confirming its spatial consistency. Furthermore, the model is validated on a practical NZTO solid electrolyte, demonstrating the broader applicability of the proposed mechanism.

This work shows that AFM feedback loop delay and frequency‐tracking limitations can induce variations in tip loading force, which in turn modulate the contact stiffness and shift the contact resonance frequency. This process gives rise to topographic crosstalk in ESM signals. The analysis demonstrates that topographic crosstalk is inherent to DART‐mode ESM measurements and provides a concrete, experimentally relevant example of the mechanism by which such crosstalk arises, together with a quantitative reference for its magnitude. These findings help prevent misinterpretation of grain‐boundary electrochemistry in battery materials. Furthermore, an optimized sample‐preparation protocol based on cooling cross‐section polishing (CCP) is proposed, substantially minimizing ESM topographic crosstalk and improving measurement reliability.

## 2 Results and Discussions

### 2.1 Detecting Topographic Crosstalk Via Trace–Retrace Scan

**FIGURE 1:**
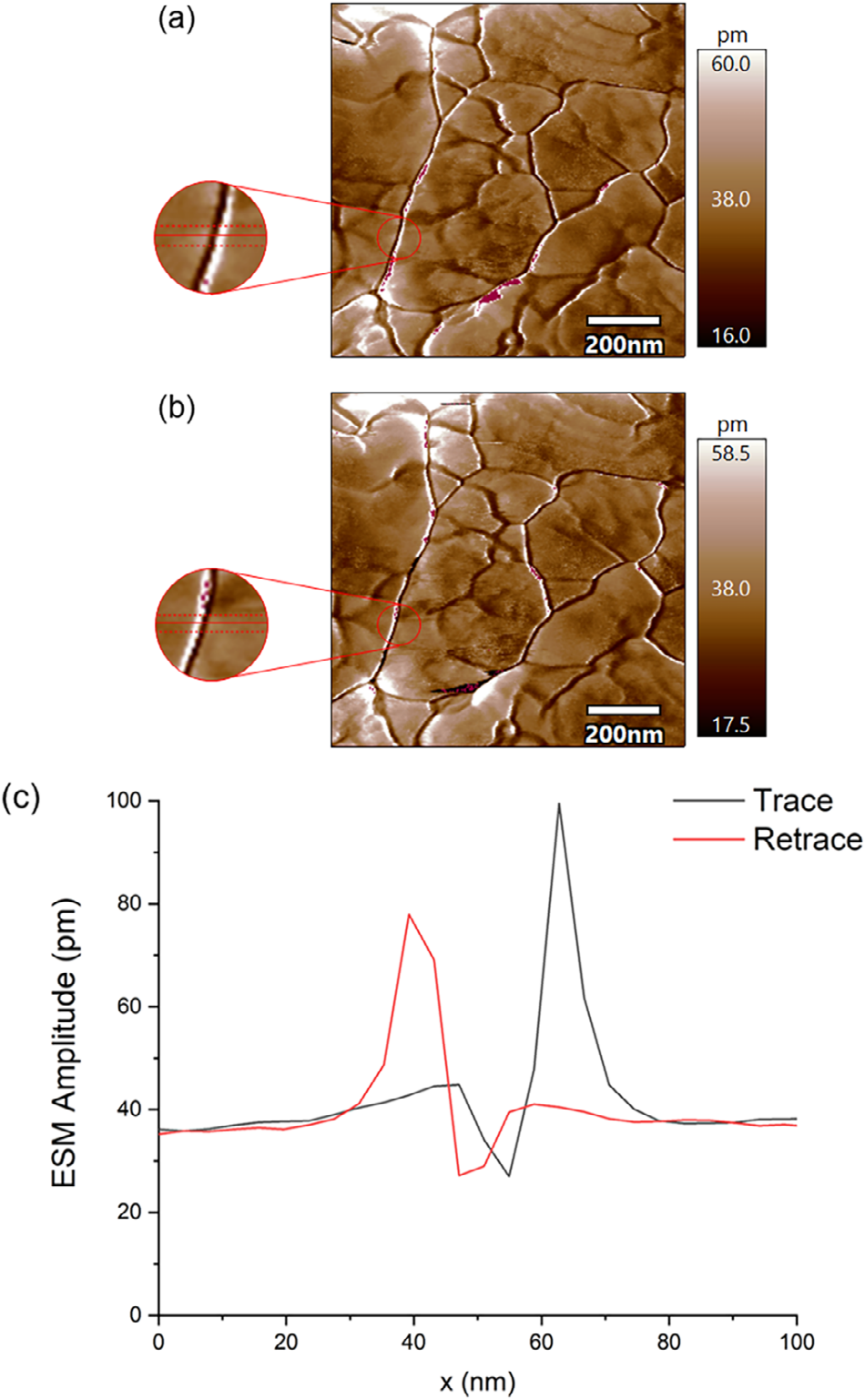
ESM amplitude of a graphite anode for lithium‐ion batteries: (a) amplitude trace, (b) amplitude retrace, and (c) line profile at the grain boundary. All ESM images presented in this work are obtained after SHO calculation to recover the true signal amplitude from the resonance‐enhanced response. Red pixels indicate regions where the SHO calculation yields errors. The full line profile is provided in Figure S1.

During ESM imaging of battery materials, distinct ESM response can be observed at grain boundaries, as shown in Figure 1. Comparing the line profiles of trace and retrace images of a graphite anode for lithium‐ion batteries across the grain boundary, the ESM signal exhibits opposite trends. This result clearly shows that ESM signal is not solely determined by the ionic concentration but also affected by artifacts that should be identified and corrected for accurately visualizing the true ionic distribution of the target material.

**FIGURE 2:**
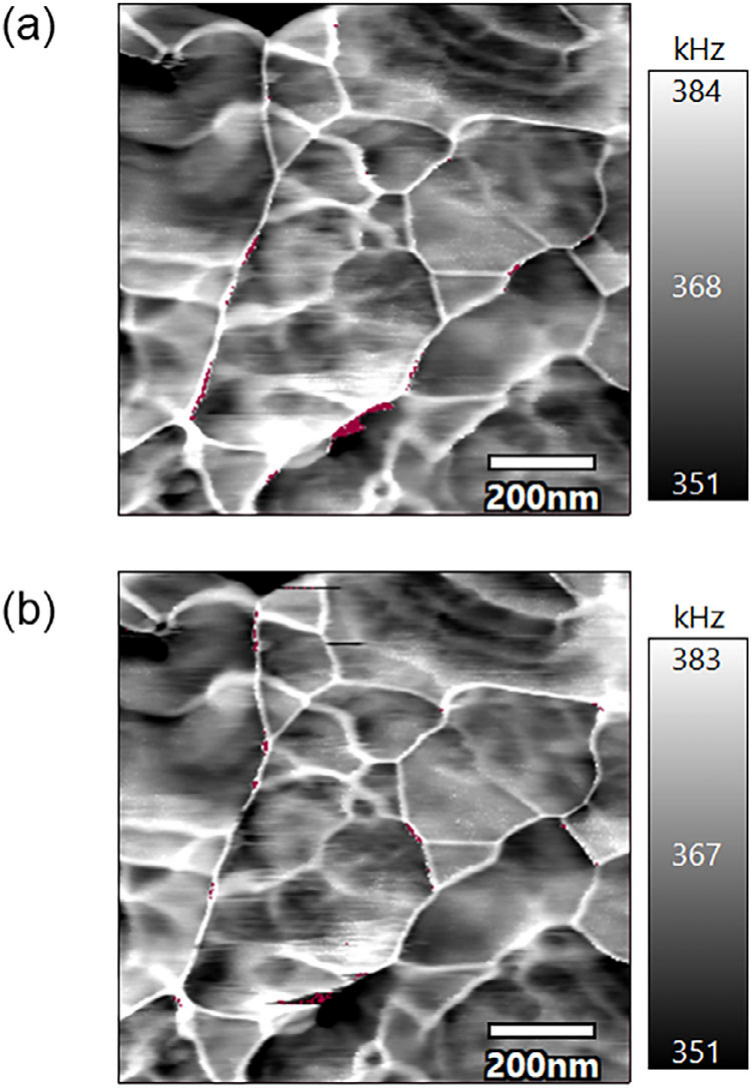
Contact resonance frequency of a graphite anode for lithium‐ion batteries: (a) trace and (b) retrace.

This crosstalk is found to originate primarily from frequency tracking delays in DART mode. The trade‐off between tracking stability and scan speed in DART‐mode measurements, as well as the potential for tracking failure under rapidly varying contact resonance conditions, has also been discussed in previous studies [30, 32, 39]. As shown in Figure 2, the AFM tip's contact resonance frequency increases at grain boundaries, resulting in significant frequency shifts as the tip scans across these features. Although the AFM continuously tracks the contact resonance frequency, inherent feedback delays produce transient amplitude variations, thereby introducing artifacts in ESM images. A detailed description of the frequency‐tracking delay and the resulting amplitude artifacts is provided in Figure S2.

**FIGURE 3:**
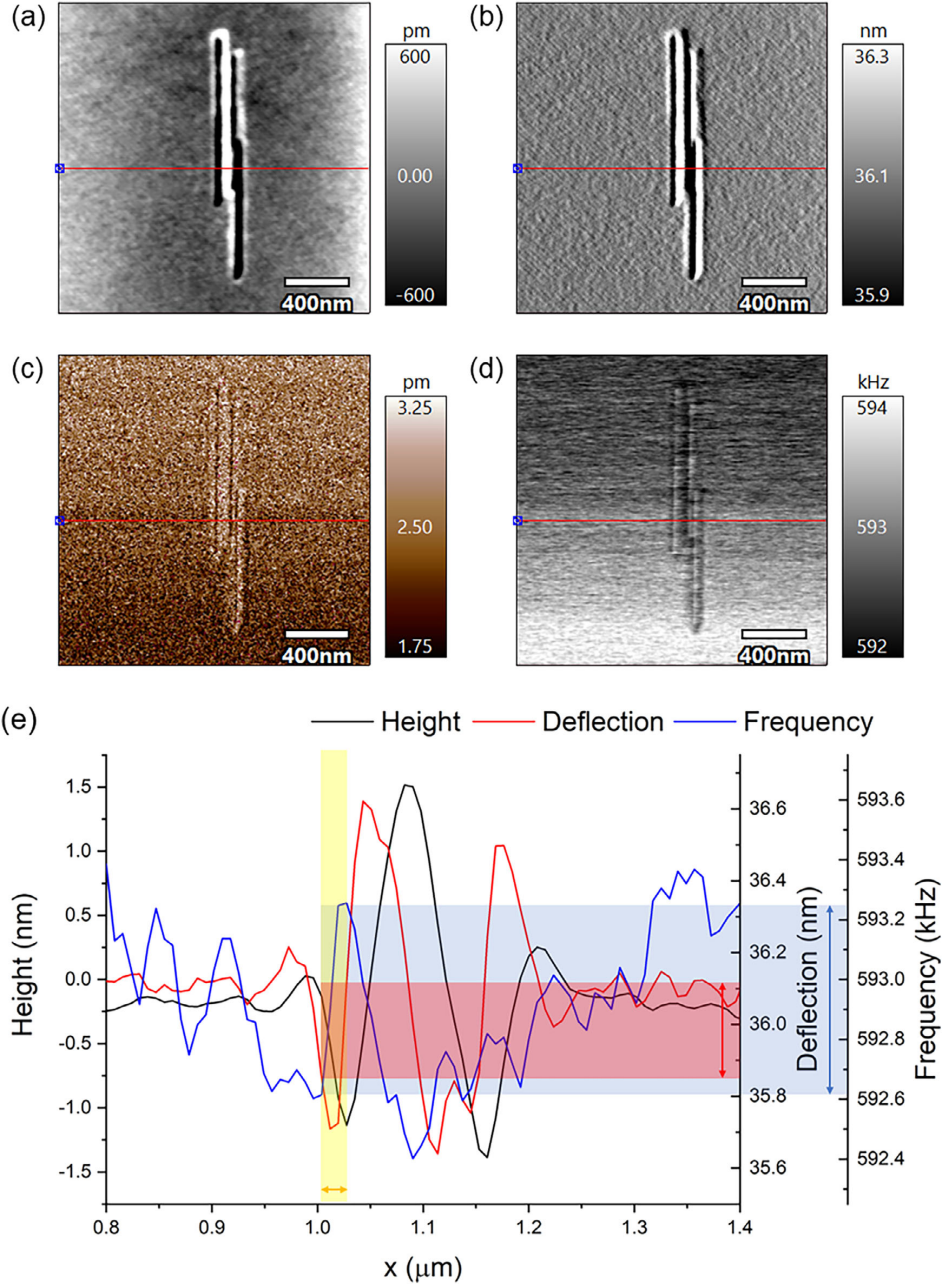
AFM images of a silicon wafer with trenches: (a) topography, (b) deflection, (c) ESM amplitude, and (d) contact resonance frequency. (e) Line profile of the AFM signals. The blue area represents the range of frequency change, the yellow area highlights the region where the frequency increases, and the red area indicates the corresponding magnitude of deflection change.

Because the high resonance frequency area corresponds to a region with significant topography variation (Figure S3), surface roughness is interpreted as a primary factor contributing to the frequency shifts and, consequently, the artifacts of the ESM image. To examine how the frequency and amplitude signals respond to topographical variations, trenches resembling grain‐boundary morphology were created on a single crystal silicon (100) wafer by scratching with a diamond‐coated AFM tip, followed by ESM measurements as shown in Figure 3. For clarity, additional line‐profile comparisons of height, ESM amplitude, and contact resonance frequency are provided in Figure S4.

Since single crystal silicon (100) wafer does not contain any mobile ions or dopants that can diffuse through the lattice, only a uniformly low ESM signal unrelated to ionic motion is expected. However, a clear ESM amplitude response exceeding the background noise level is observed on the silicon wafer, consistent with previous reports of non‐zero PFM amplitudes on silicon [40]. In addition, pronounced ESM amplitude and contact resonance frequency fluctuations are observed at the trench. These results demonstrate that ESM is not solely governed by ionic concentration and that topographic variations can induce significant ESM artifacts.

### 2.2 Analyzing Topographic Crosstalk Via SHO Modeling

To understand the origin of topographic crosstalk in ESM, it is first necessary to consider the principle of contact resonance. In ESM, the tip‐sample interaction can be modeled as two Kelvin–Voigt elements, each consisting of a spring and a dashpot connected in parallel, acting in the vertical and lateral direction (Figure S5) [23, 24, 27, 31, 41, 42]. The tip–sample interaction is a complex system in which both vertical and lateral contact stiffness, along with the intrinsic cantilever stiffness, must be considered [23, 41, 43, 44]. In addition, the mechanical properties and geometries of both the AFM tip and the sample strongly influence the interaction [23, 29, 41]. While the tip–sample interaction is complex, its dynamic response near resonance can be effectively captured by a damped harmonic oscillator (DHO) model, as widely adopted in previous studies [19, 23, 29, 30, 32, 41, 43, 45, 46, 47]. The equation of motion for the damped harmonic oscillator is given by [47]

(1) $$m\ddot{z}+\left(\frac{m\omega_{0}}{Q}\right)\dot{z}+m\omega_{0}^{2}z=F_{0}\;cos\left(\omega_{d}t\right)$$

Here, *m* denotes the effective mass of the cantilever, *z* the cantilever displacement, *ω*_0_ the resonance frequency of the oscillator, and *Q* the quality factor representing energy dissipation in the system. *F_0_* and *ω*_d_ denote the amplitude and frequency of the sinusoidal driving force applied to excite the cantilever, respectively, and *t* denotes time. The amplitude of the oscillator vibration *A_0_* can be expressed as follows: [19, 23, 29, 47]

(2) $$A_{0}=\frac{F_{0}/m}{\sqrt{{\left(\omega_{0}^{2}-\omega_{d}^{2}\right)}^{2}+{\left(\omega_{0}\omega_{d}/Q\right)}^{2}\;}}\;$$

In this work, the analysis focuses on separating and quantifying the contribution of contact stiffness variation arising from changes in tip loading force caused by AFM feedback loop delay. Given the complexity of the coupled tip–sample interaction, a simplified model is adopted to capture the dominant effect. Accordingly, damping is assumed to play a secondary role under the present experimental conditions, and all contributions to contact stiffness are incorporated into a single effective contact stiffness term for analytical clarity. Consistent with the behavior of a simple harmonic oscillator, the contact resonance frequency correspondingly increases as the effective contact stiffness increases.

(3) $$\omega_{0}=\frac{1}{2\pi }\;\sqrt{\frac{k_{eff}}{m}}$$

where *k_eff_* is the effective contact stiffness. To estimate the contact stiffness and its variation during scanning, the Hertzian contact model is employed [23, 43, 48, 49]. In this model, the AFM tip is assumed to be a rigid sphere in contact with a flat sample surface.

The detailed calculations are provided in the Supporting Information. Using Equations S1, S2, and S4, the contact stiffness of the tip‐sample system is derived as:

(4) $$k_{eff}=\frac{3}{2}\;{\left(\frac{16PRE^{*}}{9}\right)}^{\frac{1}{3}}$$

indicating that contact stiffness is proportional to the cube root of the loading force. According to Hooke's law, the loading force can also be written as

(5) $$P=k_{c}\;x$$

where *k_c_* is the cantilever's spring constant, and *x* is the cantilever deflection in AFM scanning. Thus, the loading force can be estimated by measuring the deflection signal.

In ESM, a feedback loop is used to maintain a constant cantilever bending, meaning that the deflection is ideally kept constant [25]. However, because the Z actuator requires a finite response time, the feedback loop cannot instantaneously follow surface height variations, resulting in deviations from the setpoint [50]. As shown in Figure S6, the deflection signal closely resembles the spatial derivative of the topography, consistent with previous reports [51, 52]. This behavior indicates that the cantilever deflection reflects tracking error associated with local topographic gradients, thereby giving rise to variations in the tip loading force.

Based on this, by analyzing the deflection signal, variations in contact stiffness caused by loading force variation can be tracked during scanning. Figure 3e presents the line profile of the AFM signals, showing first a decrease and then an increase in deflection as the tip scans over a trench. Based on the previously discussed principles, this indicates a first decrease and then increase in the loading force, leading to first lower and then higher contact stiffness and a corresponding fall and rise in contact resonance frequency. Although silicon does not exhibit a contact‐resonance peak associated with ionic motion, the DART frequency‐tracking algorithm still identified a peak corresponding to the purely mechanical contact resonance, which appears with a much lower amplitude. Due to frequency tracking delay, this rapid frequency increase caused a temporary amplitude reduction followed by a sharp increase. Consequently, it can be concluded that variations in contact stiffness contribute to the topographic crosstalk observed in ESM. Furthermore, as illustrated in Figure S7, a slight increase in the Q‐factor is observed when the tip enters the trench region, providing additional evidence that local contact stiffness increases upon the tip's interaction with the trench.

The quantitative relationship between loading force (deflection), contact stiffness, and contact resonance frequency can be verified locally. The measured resonance frequency increases from 592.615 to 593.256 kHz, corresponding to a relative change of:

(6) $$\frac{\Delta \omega }{\omega }=\frac{593.256kHz-592.615kHz}{592.615kHz}≅0.11\%$$

Using Equations (3), (4), (5), the dependence of contact resonance frequency on tip deflection can be summarized as, considering only the effect of contact stiffness variation:

(7) $$\omega_{0}=\frac{1}{2\pi }\;{\left[\frac{3}{2m}{\left(\frac{16Rk_{c}E^{*}}{9}\right)}^{\frac{1}{3}}\right]}^{\frac{1}{2}}\cdot x^{\frac{1}{6}}$$

Therefore, using *T* to denote the trench and *S* to denote the silicon wafer, expected relative frequency change can be calculated as follows:

(8) $$\frac{\Delta \omega_{expected}}{\omega_{S}}={\left(\frac{x_{T}}{x_{S}}\right)}^{\frac{1}{6}}-1={\left(\frac{36.1183}{35.8506}\right)}^{\frac{1}{6}}-1≅0.12\%$$

Since the calculated relative frequency change (0.12%) closely matches the actual frequency increase (0.11%), this consistency validates the reliability of our interpretation.

**FIGURE 4:**
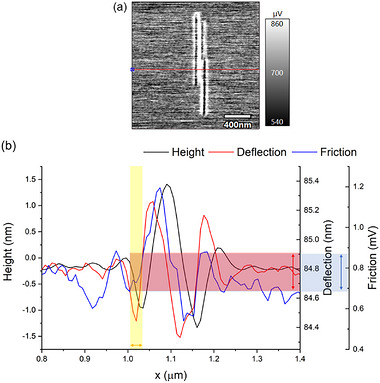
(a) Friction force map of a silicon wafer with trenches. (b) Line profiles of topography, deflection, and friction force variations in AFM signals. The yellow area corresponds to the region highlighted in Figure 3e, the red area represents the range of deflection change, and the blue area indicates the corresponding magnitude of friction change.

To verify that the loading force increases at the trench, friction force was measured, as shown in Figure 4. According to the equation

(9) $$F_{f}=\mu F_{N}$$

where *F_f_* is the friction force, *µ* is the coefficient of friction, and *F_N_* is the normal force applied to the sample surface, friction force is proportional to the normal force. Assuming that *F_N_* corresponds to the loading force *P* and that the coefficient of friction *µ* is constant, changes in friction can be used to estimate variations in the loading force. The line profile in Figure 4b shows that the friction force exhibits a trend similar to the deflection signal, both increasing as the tip encounters the trench, even along the downhill slope. This confirms that the loading force is indeed higher at the trench, providing additional validation for our interpretation.

To further quantify the accuracy of the model, frequency and deflection changes were measured at ten evenly spaced locations along the trench. The resulting mean absolute error between the calculated relative frequency change and the experimentally observed frequency increase is 0.18%. In addition, the maximum ESM amplitude increase induced by topographic crosstalk on the trenched silicon wafer reaches 69.17%, increasing from 2.40 pm on the flat region to 4.06 pm at the trench.

So far, the effect of contact stiffness variation, arising from loading‐force changes induced by feedback‐loop delay, has been verified locally in ESM signals. To extend this analysis, the expected variation in resonance frequency, approximated as proportional to the deflection to the 1/6 power according to Equation 7, is compared with the experimental results, with the corresponding line profile shown in Figure S8. To avoid the influence of baseline and scaling effects, the curves are normalized to the range of 0–1 prior to correlation analysis. A Pearson correlation analysis is performed between the two curves to evaluate their linear relationship, defined as [53]:

(10) $$r_{xy}=\frac{\sum_{i=1}^{n}\left(x_{i}-\bar{x}\right)\left(y_{i}-\bar{y}\right)}{\sqrt{\sum_{i=1}^{n}{\left(x_{i}-\bar{x}\right)}^{2}}\sqrt{\sum_{i=1}^{n}{\left(y_{i}-\bar{y}\right)}^{2}}}\;$$

where *n* is the number of data points and *x_i_, y_i_* are the individual sample points indexed by *i*. The resulting coefficient is low *(r* = 0.05), indicating weak linear correlation between the signals. This behavior may be attributed to peak shifts and variations in signal magnitude arising from delays in DART tracking.

In contrast, cross‐correlation analysis yields a normalized coefficient of 0.93 at zero lag. The cross‐correlation is defined as [54]:

(11) $$\rho_{xy}=\frac{\sum_{i=0}^{n}x_{i}y_{i-\tau }}{\sqrt{\sum_{i=1}^{n}{x_{i}}^{2}}\sqrt{\sum_{i=1}^{n}{y_{i-\tau }}^{2}}}\;$$

where *τ* denotes the lag between the two signals. Unlike Pearson correlation, no mean subtraction is applied in this formulation, such that the coefficient reflects structural similarity rather than linear correlation. While Pearson correlation is sensitive to variations in signal magnitude, cross‐correlation captures the spatial alignment between the deflection and frequency signals. The result indicates that the observed variations share a common topographic origin, demonstrating that the spatial distribution of the ESM response is governed by topography, even though the signal magnitude is influenced by additional factors.

To assess reproducibility and further evaluate the effect of topography‐induced artifacts, ESM measurements were also performed on different silicon AFM calibration samples (Figures S9–S11). ESM Topographic artifacts were repeatedly observed across different samples and tips, although their magnitude varied depending on local geometry and environmental conditions, such as humidity and temperature. These observations suggest that contact stiffness is not the only factor influencing the ESM signal. Nevertheless, the results support the conclusion that ESM topographic artifacts arising from contact stiffness variation, induced by loading‐force changes caused by feedback loop delay, are inherent to the measurement and can contribute significantly to the apparent ESM response, even on non‐ionic silicon substrates.

### 2.3 Eliminating Topographic Artifacts Via Cooling Cross‐sectional Polishing

A similar consideration applies to the graphite anode discussed in Figure 1. In battery materials, ionic conduction is spatially heterogeneous, and grain boundaries are often examined as potential fast transport pathways [16, 55]. Although a high ESM amplitude is observed at grain boundaries in the graphite anode (Figure 1), the present results show that this contrast can arise from not only from Li^+^ ionic transport but also from topography‐induced contact stiffening. Therefore, ESM contrast alone is insufficient to identify true ionic conduction pathways.

**FIGURE 5:**
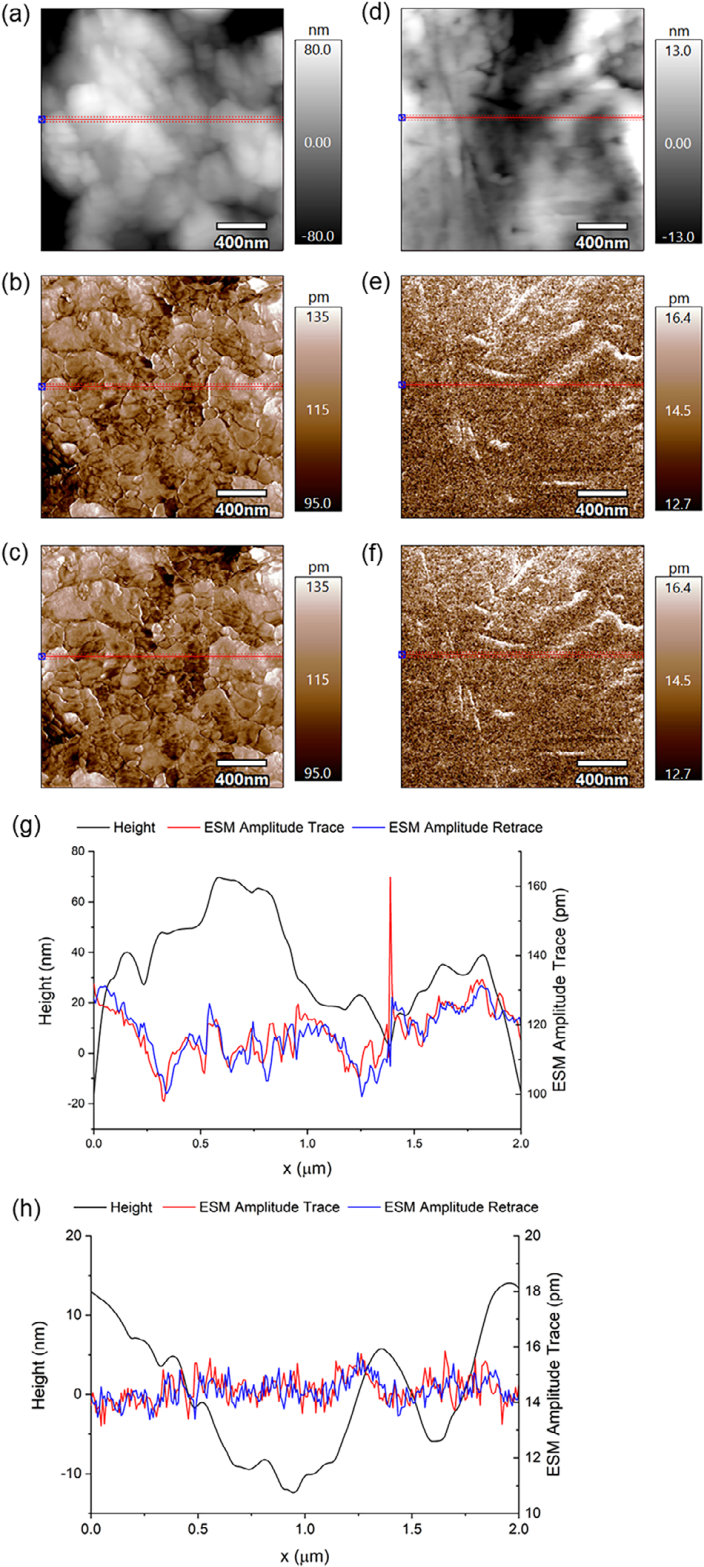
AFM images of an NZTO solid electrolyte for sodium‐ion batteries: (a–c) mechanically polished using sandpaper; (d–f) after CCP treatment. (a,d) topography, (b,e) amplitude trace, and (c,f) amplitude retrace. (g,h) Line profiles of the AFM signals comparing the consistency of amplitude trace and retrace before (g) and after (h) CCP.

Similar to observations in graphite anodes, Figure 5 demonstrates that topographic crosstalk can enhance the ESM amplitude at grain boundaries in a sodium‐ion battery solid electrolyte Na_2_Zn_2_TeO_6_ (NZTO). This spurious signal enhancement is effectively suppressed by cooling cross‐section polishing (CCP). As shown in Figure 5g,h, argon ion beam treatment in CCP markedly reduces the surface roughness of the solid electrolyte, from ∼90 to ∼25 nm, enabling a significantly smoother surface for subsequent ESM measurements.

In the sample mechanically polished using sand paper but without CCP treatment, (Figure 5b,c,g), the ESM amplitude exhibits pronounced inconsistencies between the trace and retrace images as the tip scans across rough surface regions. Localized high‐intensity signals appear when the tip passes over areas with significant topographic variation, whereas these features are absent in the opposite scan direction, indicating the presence of topographic crosstalk.

Comparison of the ESM amplitude line profile with the deflection and frequency variations in Figure S12 shows that the positions of anomalously enhanced ESM signals coincide with regions exhibiting abrupt changes in deflection and contact resonance frequency. In practical battery materials systems, the surface topography is irregular, and multiple coupled factors contribute to ESM artifacts. As a result, the local frequency variations in NZTO do not exactly coincide with the deflection changes in magnitude, unlike the behavior observed in the silicon reference samples.

A Pearson correlation analysis between the normalized expected contact resonance frequency and the normalized experimentally measured frequency (Figure S13a) yields a weak coefficient of −0.16, with a slightly larger magnitude and a sign reversal relative to the positive correlation observed for the silicon wafer reference. The small magnitude of the Pearson coefficient indicates a lack of point‐to‐point correspondence between the two signals. The negative sign suggests a weak anti‐correlated tendency in the local response, particularly under conditions of increased surface roughness in practical samples. Such behavior may be associated with Z‐actuator feedback‐loop delays or frequency tracking delays discussed previously.

To further examine the spatially shifted relationship between the two signals, cross‐correlation analysis is performed. A maximum cross‐correlation coefficient of 0.79 is obtained at a lag of −0.02, corresponding to a spatial shift of approximately −40 nm in a 2 µm ESM line profile with 256 data points. This indicates that the two signals exhibit similar spatial trends when a finite spatial offset is considered. These results suggest that variations in contact stiffness induced by loading‐force changes associated with feedback‐loop delay may contribute to the observed response, although their effects are likely intertwined with other topographic or instrumental artifacts.

By comparison, after CCP treatment, the trace and retrace ESM amplitude images (Figure 5e,f,h) show substantially improved agreement. Although complete overlap is not achieved, the signals become more uniform across the entire image, and most topography‐correlated variations in ESM amplitude are removed. The standard deviation of the ESM amplitude decreases from 8.36 pm before CCP to 0.93 pm after CCP, further confirming that surface smoothing via CCP effectively suppresses topographic crosstalk.

The same correlation analysis is performed on NZTO after CCP. A more negative Pearson coefficient of −0.32 and a similar cross‐correlation coefficient of 0.79 at a lag of −0.02 are obtained. The increased magnitude of the negative Pearson coefficient is likely due to the reduced signal variance after CCP, which makes the metric more sensitive to residual noise and small point‐to‐point mismatches than to overall structural correspondence. Although CCP significantly reduces surface roughness, the surface is not perfectly flat, and residual topography‐related contributions may still persist in the ESM response. Accordingly, the cross‐correlation remains largely unchanged compared to the pre‐CCP case, indicating that the underlying spatially shifted relationship between the two signals is preserved. These results suggest that the frequency variation is still influenced by residual topographic features rather than being governed by the absolute roughness magnitude.

Notably, once surface‐induced mechanical artifacts are eliminated, grain boundaries in NZTO do not exhibit an enhanced ionic response detectable by DART‐mode ESM, suggesting that they are less likely to act as fast Na^+^ ion conduction pathways. However, the specific ionic transport pathways within NZTO cannot be unambiguously resolved based on the present measurements alone. Consequently, identification of the dominant ionic transport pathways will require complementary characterization approaches beyond the scope of the present study.

## 3 Conclusions

This work identified and explicitly demonstrated a pathway by which topographic crosstalk arises from loading‐force variations induced by feedback‐loop delay in ESM measurements. An ionically inactive silicon wafer with artificial trenches, fabricated using a diamond‐coated AFM tip, was employed as a model system. Using an SHO framework, a quantitative relationship between contact resonance frequency shift and tip deflection was established. The resulting frequency variation was shown to scale approximately with the deflection raised to the 1/6 power, leading to an apparent ESM amplitude enhancement of up to 69.17% in the present silicon‐based measurements.

This mechanism was further validated in the practical sodium‐ion solid electrolyte NZTO. Pearson and cross‐correlation analyses were conducted to investigate the relationship between deflection and contact resonance frequency in the sample. These results provide a practical framework for identifying such artifacts and avoiding misinterpretation of topography‐correlated signals as intrinsic electrochemical responses. Although the magnitude of this effect depends on the specific system and measurement conditions, the silicon‐based model presented here offers an experimentally grounded reference for evaluating the potential extent of such crosstalk.

Furthermore, this work demonstrated that surface‐induced mechanical artifacts can be effectively suppressed by CCP. After polishing, grain boundaries in NZTO no longer exhibited enhanced ESM responses. This finding highlights that ESM amplitude contrast alone is insufficient for reliably identifying ionic conduction pathways, particularly in the presence of rough topography. Overall, this work emphasizes the need for careful artifact assessment for reliable interpretation of nanoscale ionic transport in battery materials.

## 4 Experimental Section

### 4.1 Graphite Anode

The electrode was fabricated by casting a slurry containing active material (96 wt.%), polymeric binders (1.5 wt.% styrene butadiene rubber and 1.5 wt.% carboxymethyl cellulose), and conductive agent (Super‐P, 1 wt.%) onto copper foil (25 µm thickness). A conventional LiPF_6_‐based EC/DMC electrolyte with FEC additive was used for electrochemical cycling of graphite‐containing cells.

### 4.2 Silicon Wafer

A single‐crystal Si(100) wafer without doping was used as a reference sample for the ESM measurements.

### 4.3 Solid Electrolyte NZTO

The sodium layered solid‐state electrolyte Na_2_Zn_2_TeO_6_ (NZTO) was synthesized via a conventional solid‐state reaction method. Na_2_CO_3_ (≥99.5%, Sigma‐Aldrich, USA), TeO_2_ (99.995%, trace metal basis, Sigma‐Aldrich, USA), and ZnO (99.99% purity, Sigma‐Aldrich, USA) were used as precursor materials for NZTO synthesis. To ensure sufficient solid‐state reaction among the precursor materials, the powders were mixed for 12 h at 450 rpm using a three‐dimensional ball‐milling system (Nano Fine Tech, Republic of Korea). After the 3D ball‐milling process, the mixed precursor powders were sintered at 800°C for 15 h in a muffle furnace using an alumina crucible. The resulting sintered powders were then pelletized and subjected to a second sintering step at 800°C for 12 h in a muffle furnace. The NZTO sample without CCP treatment was mechanically polished using 1000‐ and 2000‐grit sandpaper prior to measurements.

### 4.4 Electrochemical Strain Microscopy (ESM) Measurement

Electrochemical Strain Microscopy (ESM) is an Atomic Force Microscopy (AFM)‐based technique for measuring local ionic concentrations and ionic conduction at the nanoscale. In this technique, a periodic AC voltage is applied to the AFM tip to concentrate an electric field within a nanoscale volume of the sample. The localized electric field electrochemically induces changes in the chemical composition, leading to deformation of the sample surface. This deformation, caused by concentrated ions under the applied electric field, is referred to as Vegard strain. Higher ionic concentrations result in larger Vegard strain, which produces greater cantilever deflection. ESM measurements were performed using an MFP‐3D Origin AFM (Asylum Research, Oxford Instruments, USA) for the graphite anode and silicon wafer, and a Cypher ES Environmental AFM system (Asylum Research, Oxford Instruments, USA) for the NZTO solid electrolyte. To enhance the signal‐to‐noise ratio during ESM measurements, dual AC resonance tracking (DART) mode was employed. Signal processing was conducted using the simple harmonic oscillator (SHO) model to restore amplified signals to their original scales, with calculations performed automatically by Asylum Research software. Different AFM tips were selected according to the specific sample requirements. For graphite anode measurements, PPP‐EFM tips (Nanosensors, Switzerland) with a force constant of 2.8 N m^−1^ were used to measure the ESM amplitude response. These tips, featuring PtIr_5_ coating on both sides of the cantilever to enhance electrical conductivity, exhibited a resonance frequency of 45–115 kHz and a nominal tip radius of curvature <25 nm. For silicon wafer measurements, CDT‐FMR tips (Nanosensors, Switzerland) with a force constant of 6.2 N m^−1^, a resonance frequency of 65–115 kHz, and a tip radius of curvature of 100–200 nm were employed. The highly doped diamond coating of the CDT‐FMR tips increases wear resistance, preventing tip degradation during trench formation and subsequent ESM measurements. For NZTO solid electrolyte measurements, diamond‐coated CDT‐CONTR tips (Nanosensors, Switzerland) with a force constant of 0.1–1.7 N m^−1^, a resonance frequency of 11–29 kHz, and a tip radius of curvature of 100–200 nm were utilized to avoid peak‐shifting issues observed with PPP‐EFM tips on this material.

### 4.5 Cooling Cross Section Polishing (CCP)

To polish the sample surface and reduce topographical artifacts, a cooling cross section polisher (JEOL, Japan) was employed. The sample was polished using a 5 kV argon ion beam for 20 h. During the CCP process, the temperature was maintained at −120°C to minimize damage induced by the argon ion beam.

## Author Contributions

D. Chen and S. Hong conceived the study. D. Chen performed the ESM measurements and carried out the data analysis. Y. Choi, C. Gong, and A. Saha assisted with the ESM measurements. J. Lee, C. Song, S. H. Han, N.‐S. Choi, and J. M. Yuk provided the battery samples. D. Chen wrote the manuscript. S. Hong supervised the work and revised the manuscript.

## Funding

This work was supported by the National Research Foundation of Korea (NRF) grant funded by the Korea government (MSIT) (Nos. RS‐2026‐25468150 and RS‐2023‐00247245).

## Conflicts of Interest

The authors declare no conflicts of interest.

## Supporting information




**Supporting File**: smtd70763‐sup‐0001‐SuppMat.docx.

## Data Availability

The data that support the findings of this study are available from the corresponding author upon reasonable request.
