# Supplementary material for: Quantitative Analysis of Topographic Crosstalk in DART‐ESM Arising from Feedback‐Loop‐Delay‐Induced Contact Stiffness Variations in Battery Materials
Source: Small Methods. 2026 Jun 11;10(13):e70763. doi: 10.1002/smtd.70763 (PMC13353891; doi:10.1002/smtd.70763)
Supplement: Supplementary file 1 — Supporting File: smtd70763‐sup‐0001‐SuppMat.docx. [file SMTD-10-e70763-s001.docx]

Supporting Information

**Quantitative Analysis of Topographic Crosstalk in DART-ESM Arising from Feedback-Loop-Delay-Induced Contact Stiffness Variations in Battery Materials**

Dongyan Chen, Junki Lee, Chaeeun Song, Seung Hee Han, Youngwoo Choi, Chaewon Gong, Aditi Saha, Nam-Soon Choi, Jong Min Yuk, and Seungbum Hong*

D. Chen, J. Lee, Y. Choi, C. Gong, A. Saha, J. M. Yuk, S. Hong

Department of Materials Science and Engineering, Korea Advanced Institute of Science and Technology (KAIST), 34141, Daejeon, Korea

E-mail: [seungbum@kaist.ac.kr](mailto:seungbum@kaist.ac.kr)

C. Song, S. H. Han, N.-S. Choi

Department of Chemical and Biomolecular Engineering, Korea Advanced Institute of Science and Technology (KAIST), 34141, Daejeon, Korea

S. Hong

KAIST Institute for Climate, Energy, and Environment, Korea Advanced Institute of Science and Technology (KAIST), 34141, Daejeon, Republic of Korea

1. Full line profiles of the ESM amplitude measured on a graphite anode for lithium-ion batteries, comparing trace and retrace.

The characteristic patterns observed in the ESM amplitude trace and retrace in Figure 1c are consistently repeated across the entire line profile, with their positions corresponding to the locations of grain boundaries, indicating the presence of topographic artifacts.


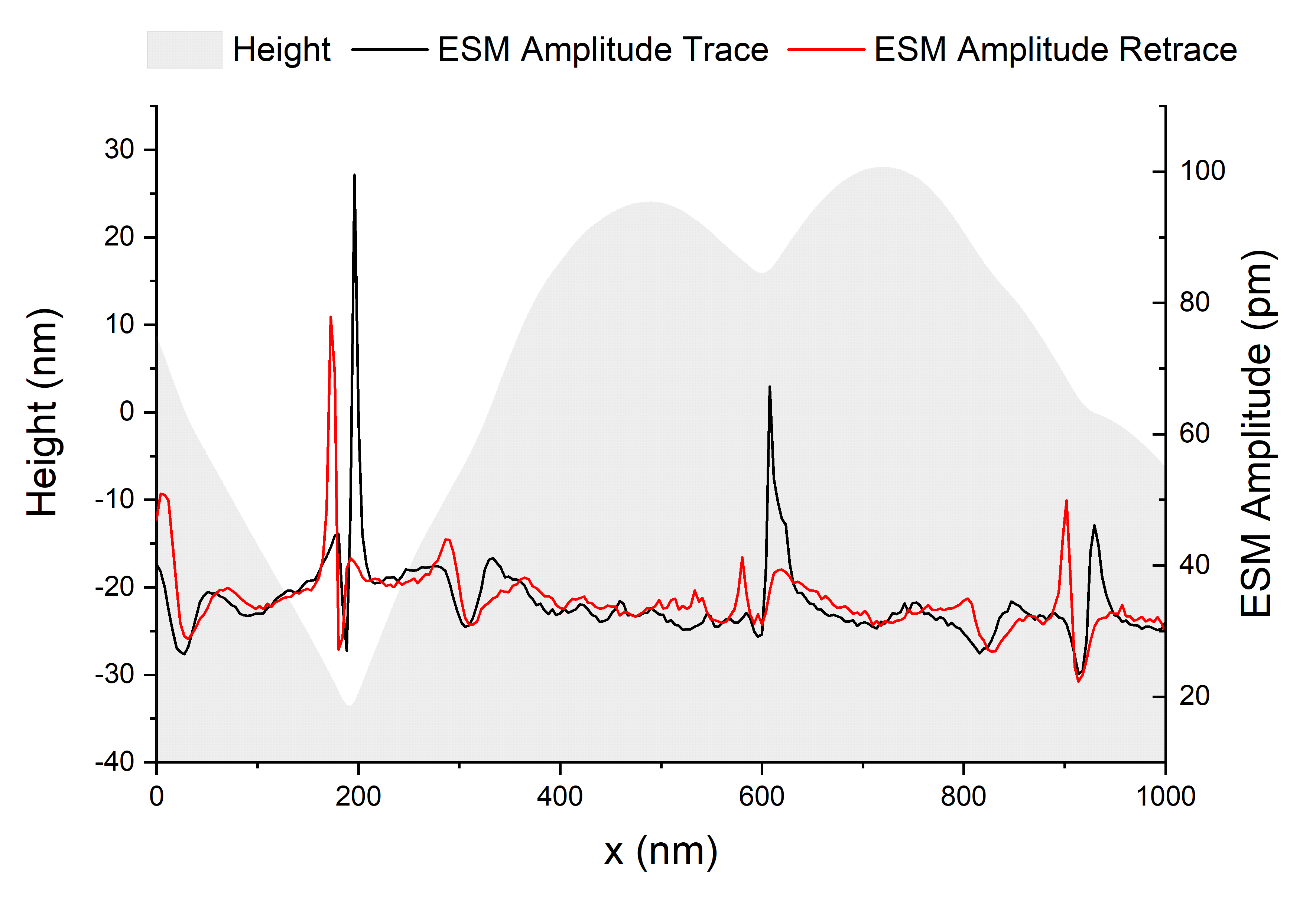


**Figure S1.** Full line profiles of the ESM amplitude measured on a graphite anode for lithium-ion batteries, comparing trace and retrace.

1. Frequency-tracking behavior in DART-mode ESM with tracking delay

When the tip crosses a grain boundary, the contact resonance frequency rises, shifting the peak rightward in the amplitude-frequency curve (**Figure S2a**). Due to the large frequency shift, the system requires time to adjust, causing a temporary amplitude 1 decrease. Once the tip moves into the grain interior, the contact resonance frequency drops, shifting the peak leftward (**Figure S2b**). The tracking delay in this transition results in an amplitude 1 increase.

Consequently, when the tip encounters a grain boundary with high resonance frequency, the amplitude 1 signal first decreases and then increases during the scan. In AFM imaging, the trace scan moves the AFM tip from left to right, while the retrace scan moves the tip from right to left. Due to the opposite scanning directions, the ESM amplitude exhibits reversed tendencies between trace and retrace images.


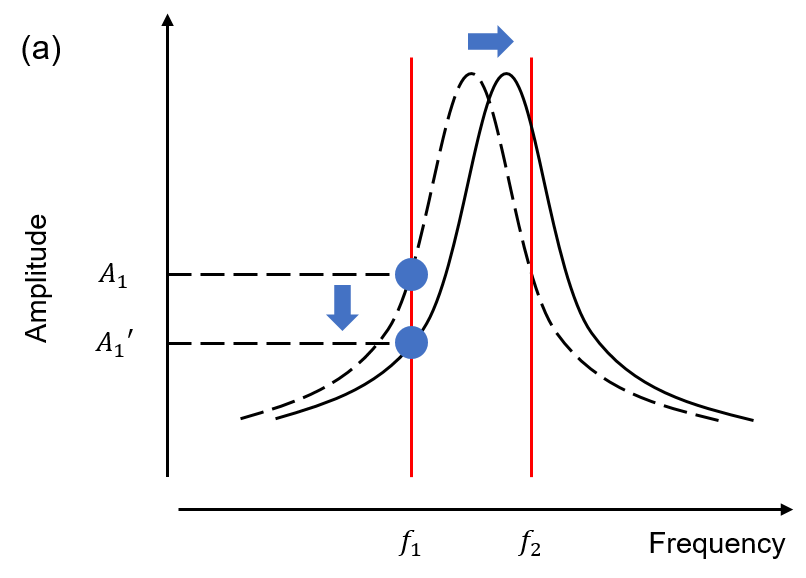


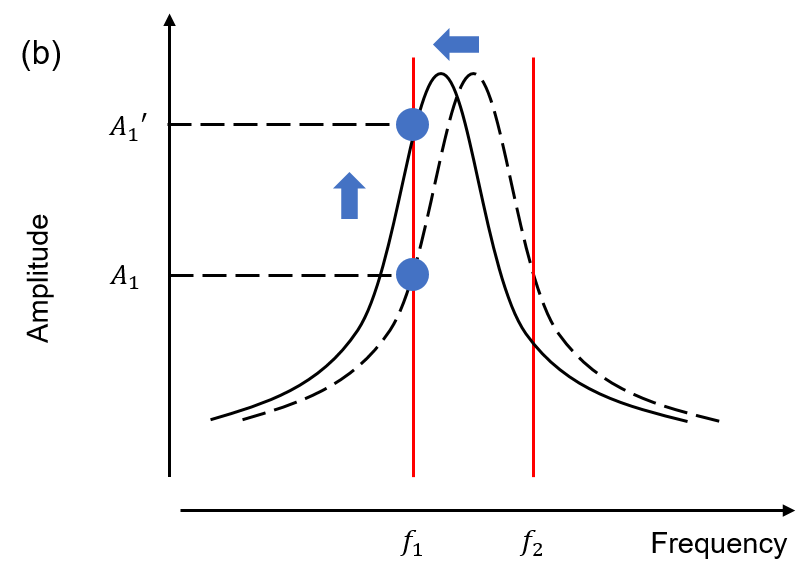


**Figure S2.** Schematic diagrams illustrating frequency-tracking behavior in DART-mode ESM under tracking delay. (a) An upward shift in the true resonance frequency leads to a lower measured amplitude 1 due to delayed tracking. (b) A downward shift in resonance frequency results in an apparent increase in amplitude 1 for the same reason.

1. Topography of a graphite anode for lithium-ion batteries


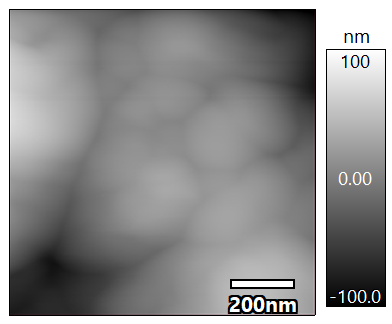


**Figure S3.** Topography of a graphite anode for lithium-ion batteries.

1. Line profiles of the AFM signals, including height, ESM amplitude, and contact resonance frequency, measured on a silicon wafer with trenches


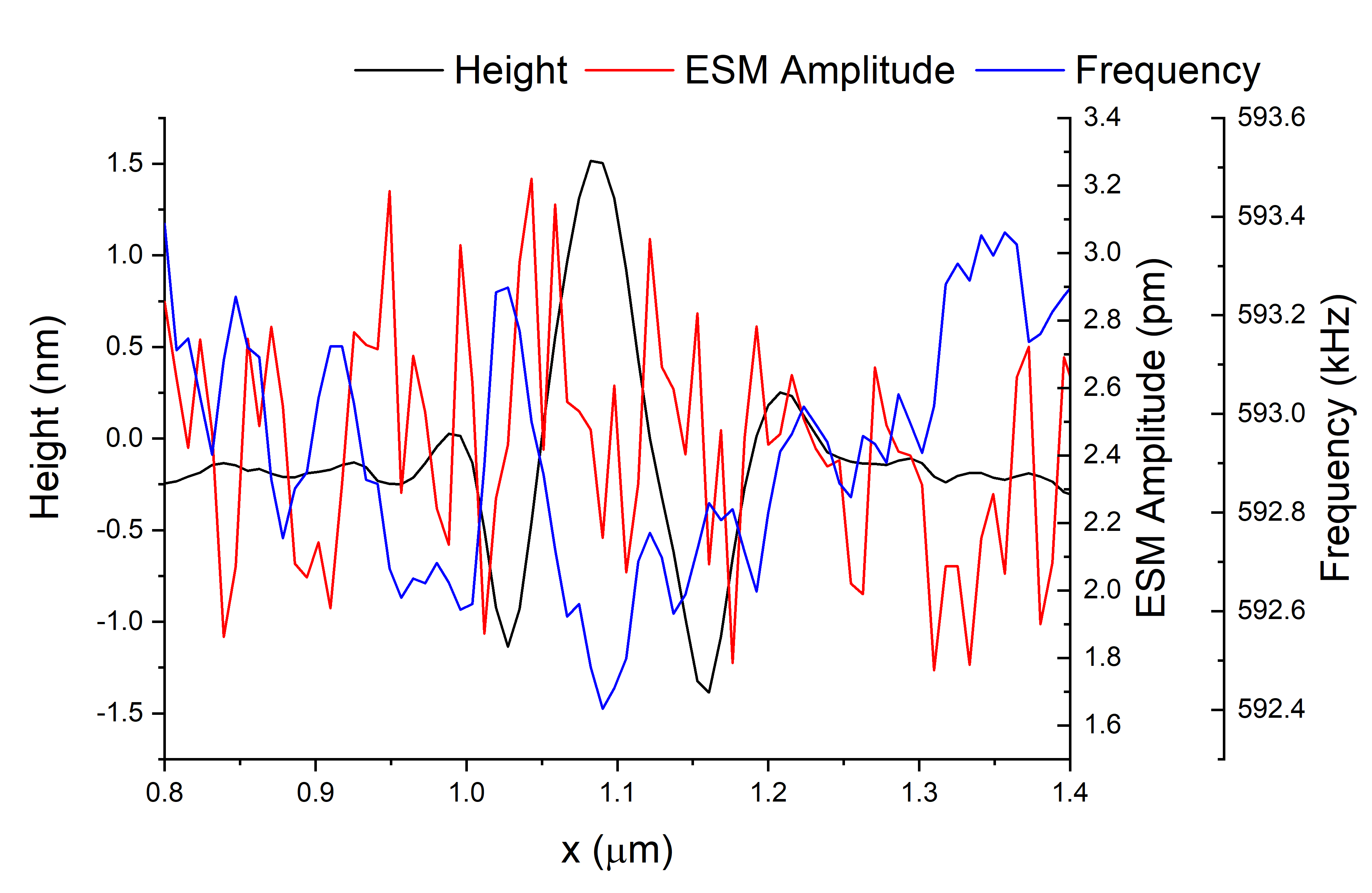


**Figure S4.** Line profiles of the AFM signals, including height, ESM amplitude, and contact resonance frequency, measured on a silicon wafer with trenches.

1. Schematic illustration of tip-sample interaction in ESM


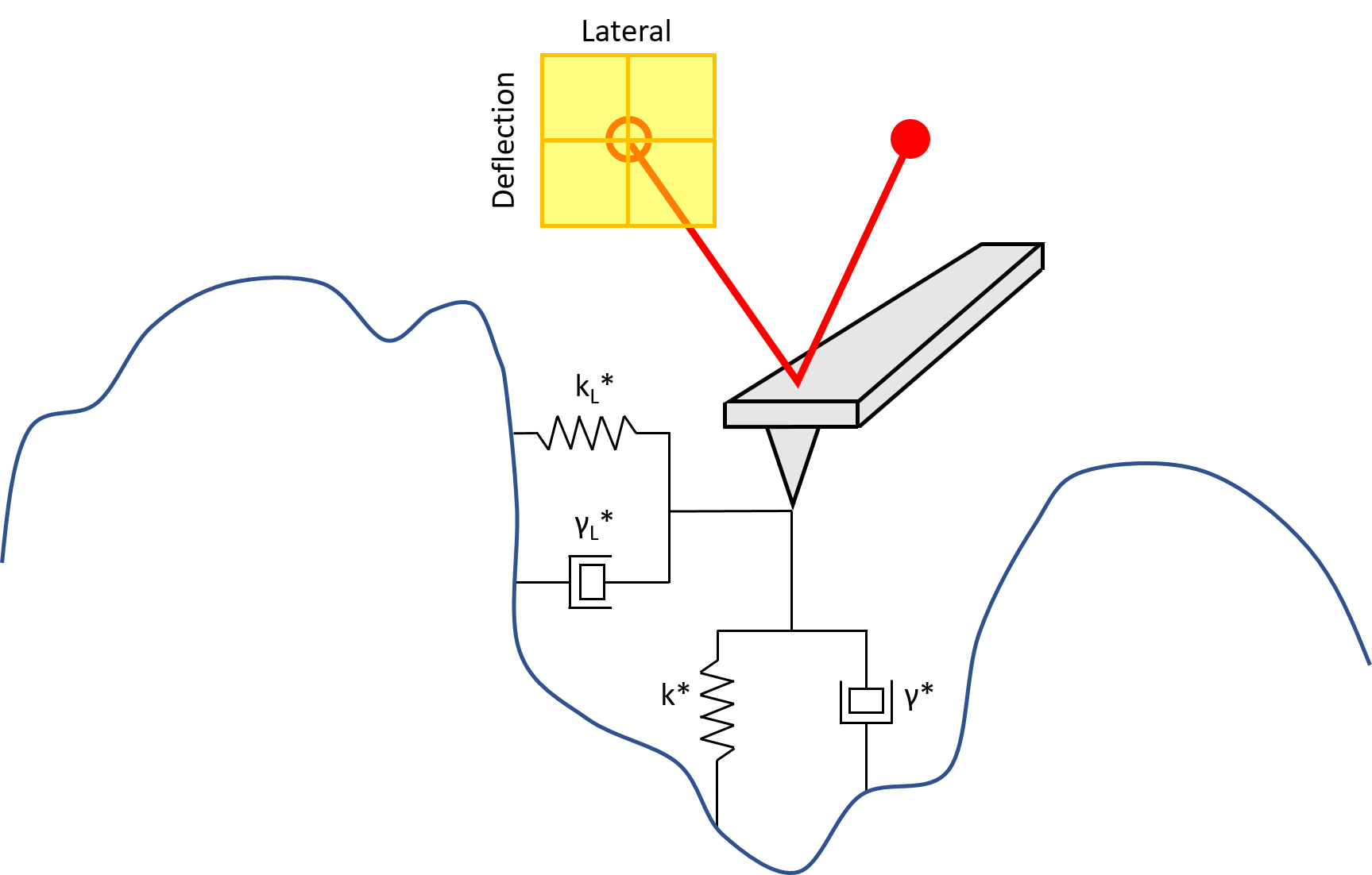


**Figure S5.** Schematic illustration of tip-sample interaction in ESM. *k^*^* denotes the normal contact stiffness and *γ^*^* represents the normal contact damping, while *k_L_^*^* and *γ_L_^*^* are the corresponding coefficients in the lateral direction.

1. Derivation of Equation 8

According to Hooke’s law, the contact stiffness is given by

| $k_{eff}=\frac{dP}{dz}$ | (S1) |
| --- | --- |

where *P* is the loading force and *z* is the tip displacement. For simplicity, the contact area is approximated as a circular region with radius *a*. The relation between *P* and *a* is expressed as

| $P=\frac{4a^{3}E^{*}}{3R}$ | (S2) |
| --- | --- |

where *R* is the radius of curvature of the AFM tip, and *E^*^* is the effective elastic modulus, defined as

| $\frac{1}{E^{*}}=\frac{\left( 1-\nu_{1}^{2} \right)}{E_{1}}+\frac{\left( 1-\nu_{2}^{2} \right)}{E_{2}}$ | (S3) |
| --- | --- |

Here *E_1_*, *E_2_* are the elastic moduli of the indenter (AFM tip) and the sample, respectively, while *ν_1_*, *ν_2_* are their Poisson’s ratios. The tip displacement *z* is given by

| $z=\frac{a^{2}}{R}=\left( \frac{9P^{2}}{16R{E^{*}}^{2}} \right)^{\frac{1}{3}}$ | (S4) |
| --- | --- |

Using Equations S1, S2, and S4, the contact stiffness of the tip–sample system is derived, as shown in Equation 8.

1. Deflection signal closely resembles the spatial derivative of the topography


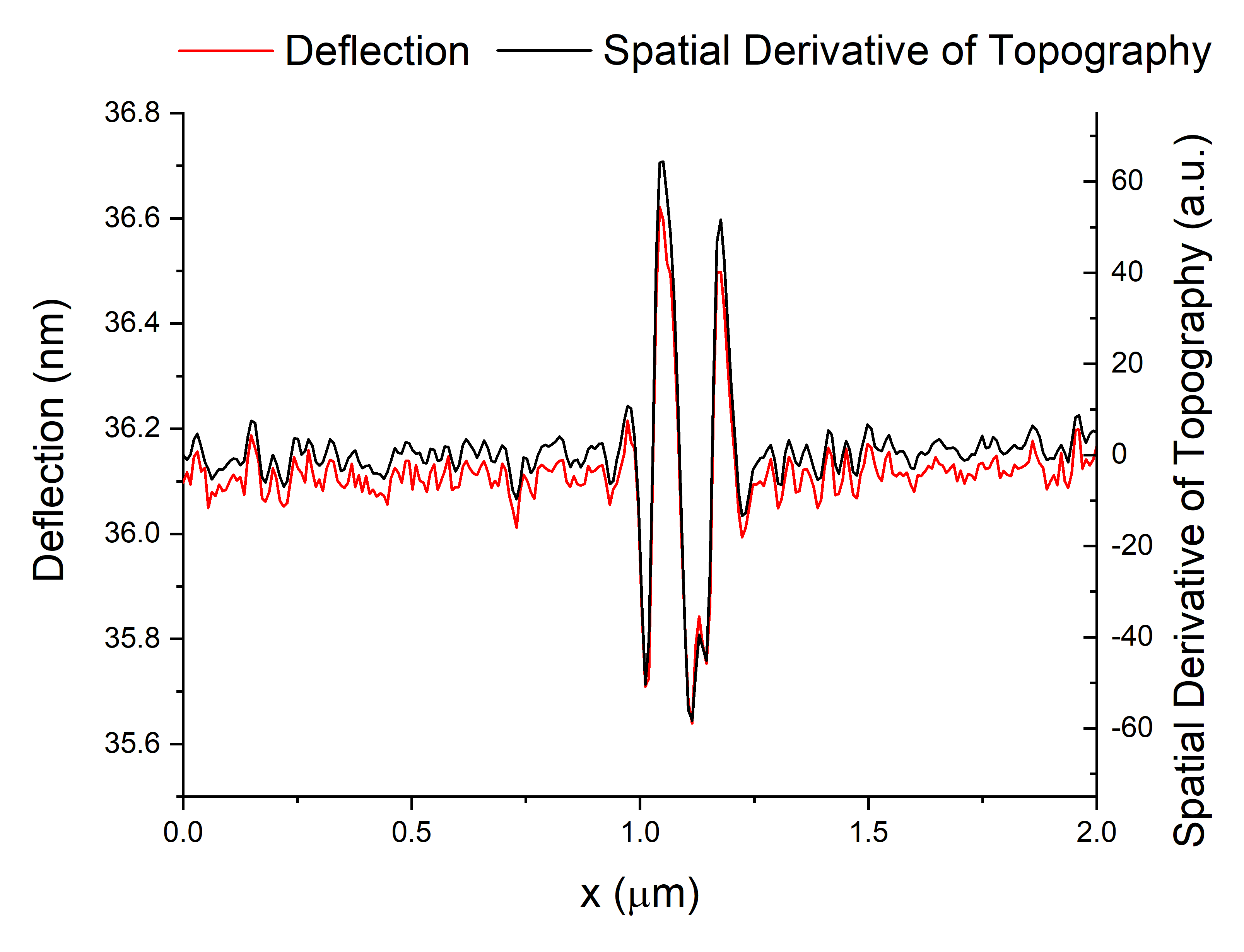


**Figure S6.** Line profiles of the AFM signals, including deflection and the spatial derivative of the topography, measured on a silicon wafer with trenches.

1. Q-factor variation measured on a silicon wafer with trenches


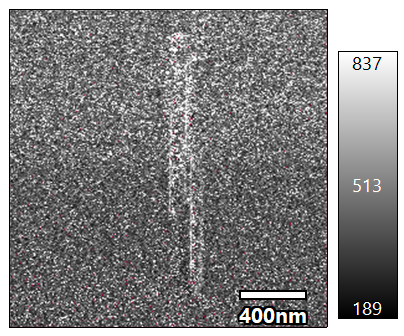


**Figure S7.** Q-factor variation measured on a silicon wafer with trenches.

1. Comparison of the normalized expected resonance frequency with the normalized experimentally measured frequency across trenches on the silicon wafer.


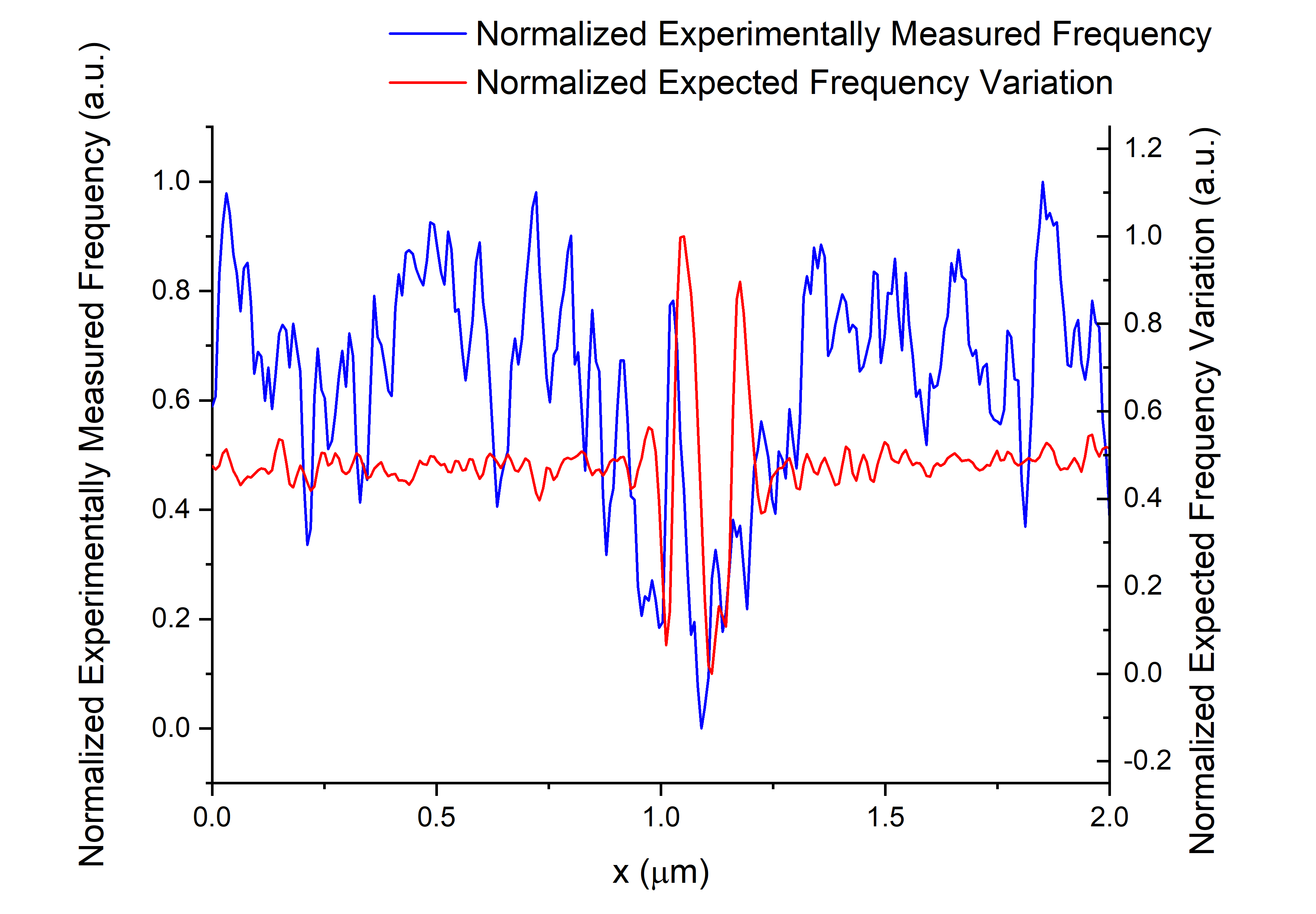


**Figure S8.** Comparison of the normalized expected resonance frequency with the normalized experimentally measured frequency across trenches on the silicon wafer.

1. ESM topographic crosstalk on silicon calibration samples


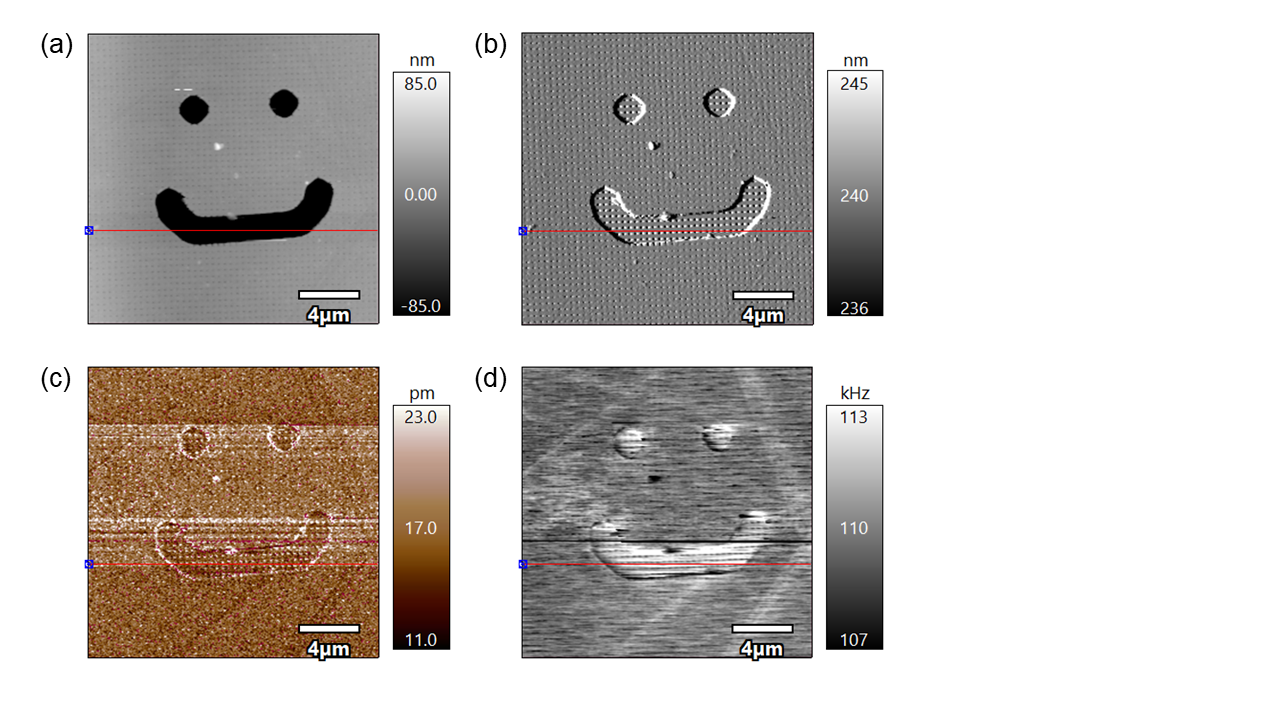


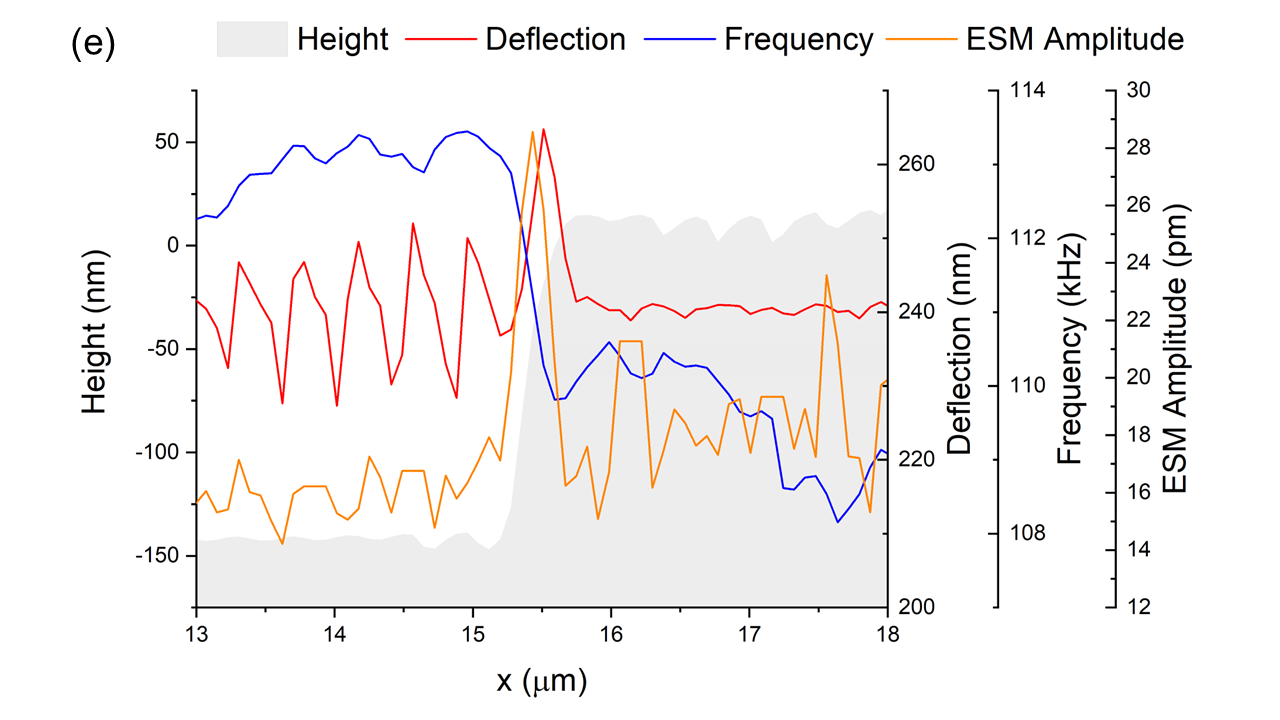


**Figure S9.** AFM images of a silicon AFM calibration grating (AFM Fun Grating, Nanosensors, Switzerland) with smile-shaped topographic features: (a) topography, (b) deflection, (c) ESM amplitude, and (d) contact-resonance frequency. (e) Line profile of the AFM signals.


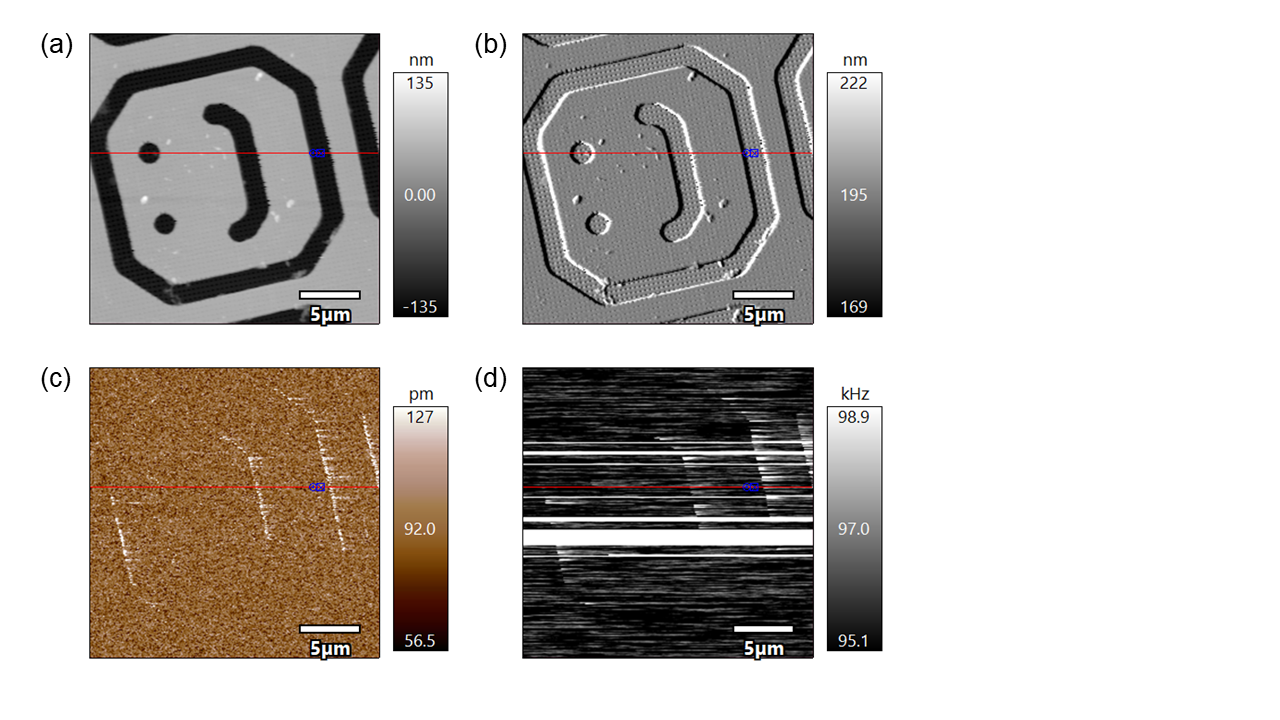


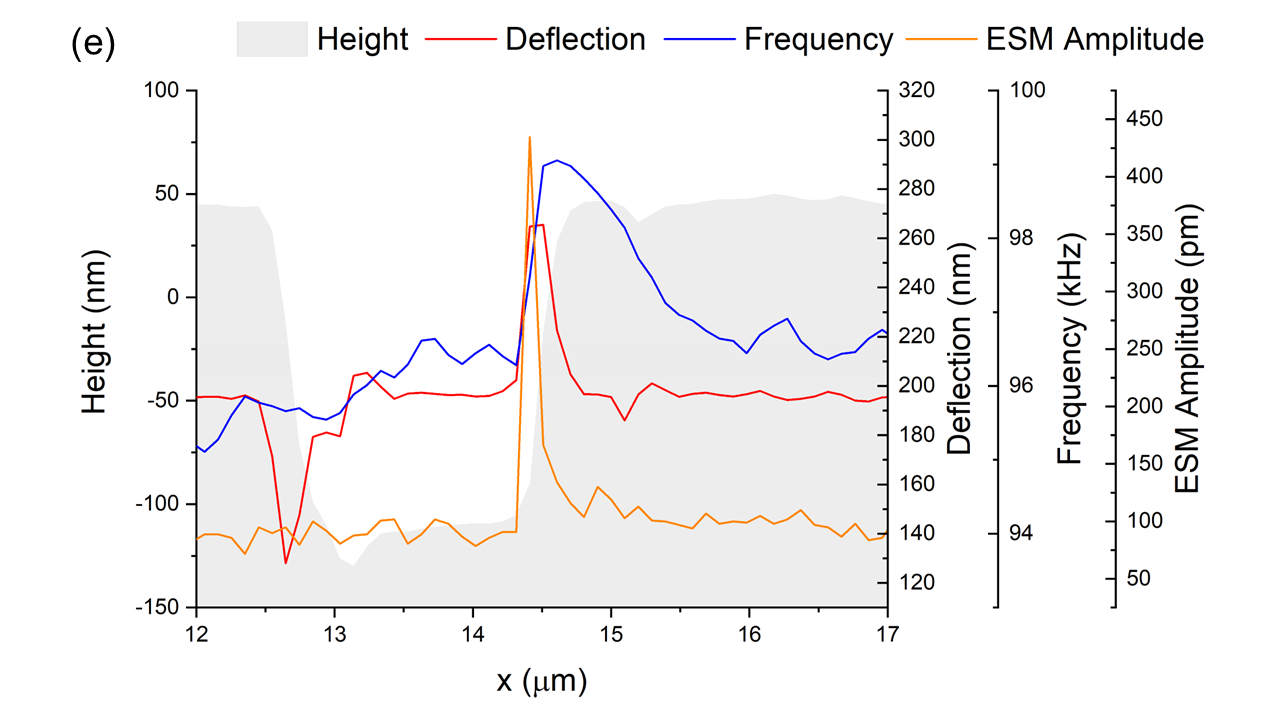


**Figure S10.** AFM images acquired on the same silicon AFM calibration grating (AFM Fun Grating, Nanosensors, Switzerland) during a separate measurement session, demonstrating reproducibility: (a) topography, (b) deflection, (c) ESM amplitude, and (d) contact-resonance frequency. (e) Line profile of the AFM signals.


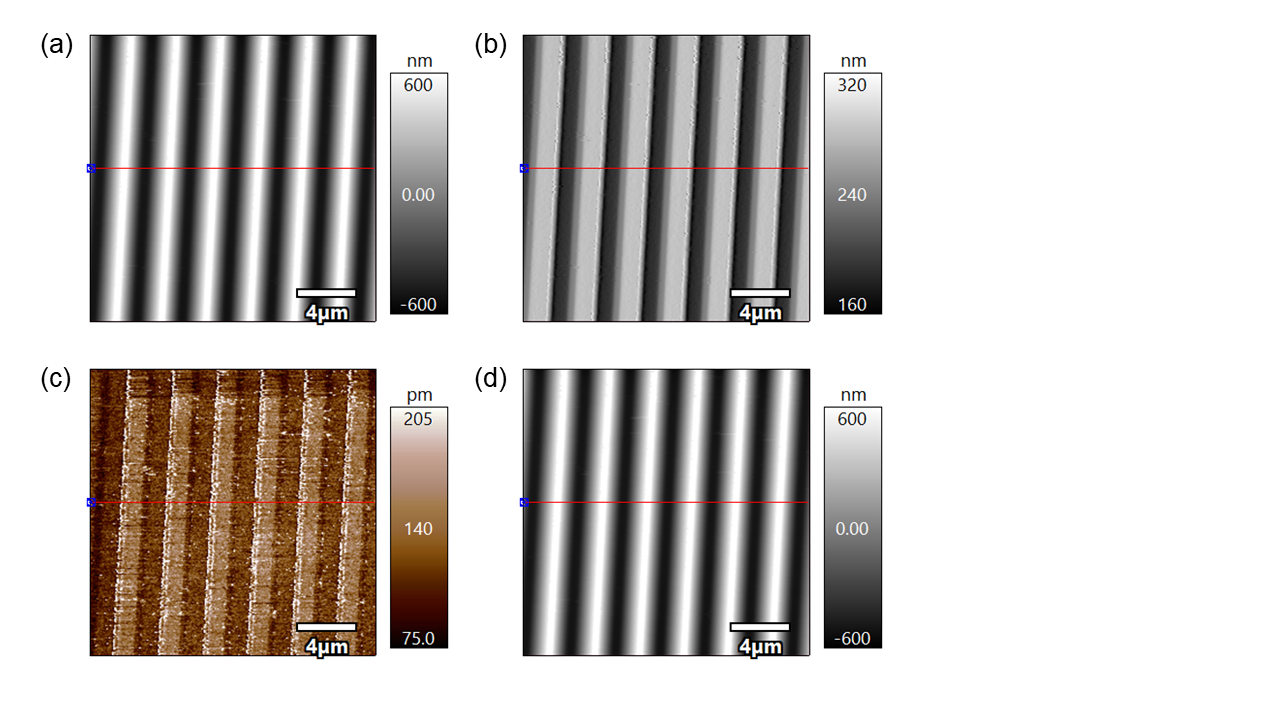


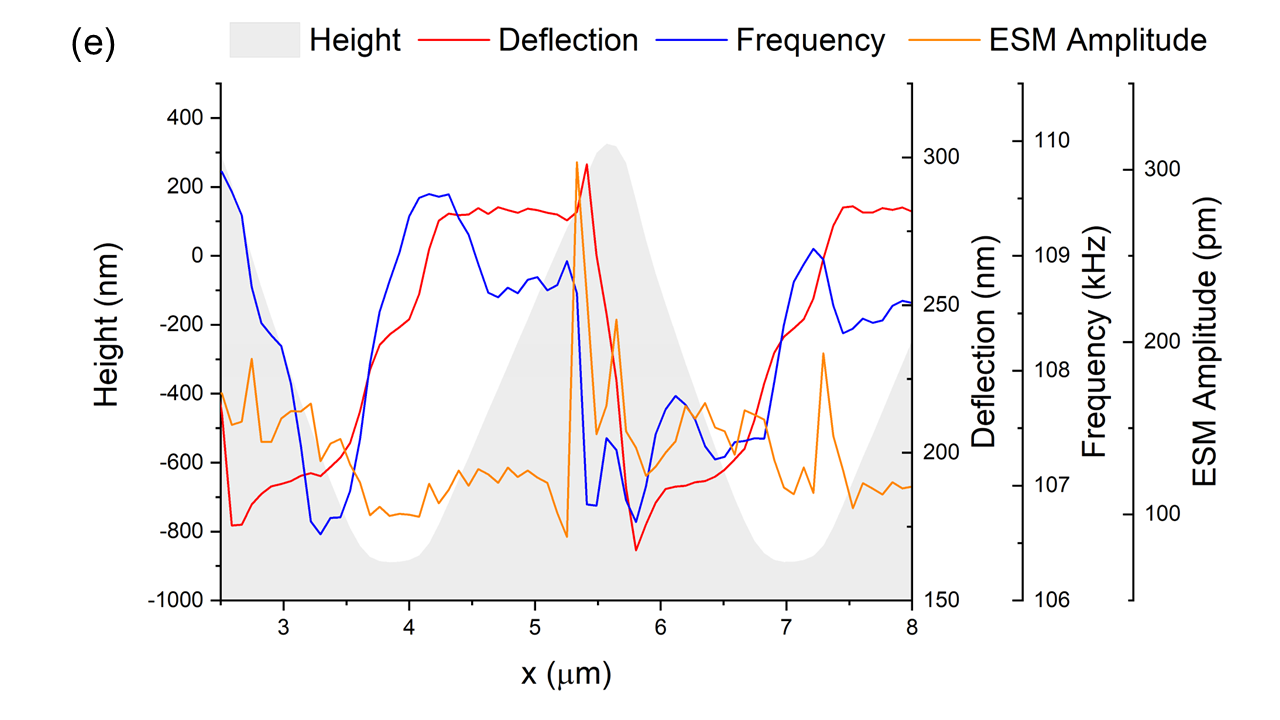


**Figure S11.** AFM images of a silicon AFM calibration grating (TGG1, Scansens, Germany) with a one-dimensional array of triangular steps: (a) topography, (b) deflection, (c) ESM amplitude (without SHO calculation), and (d) contact-resonance frequency.

1. Line profiles of the AFM signals, including height, deflection, contact resonance frequency, and ESM amplitude, measured on NZTO solid electrolyte under sandpaper polishing and after CCP treatment


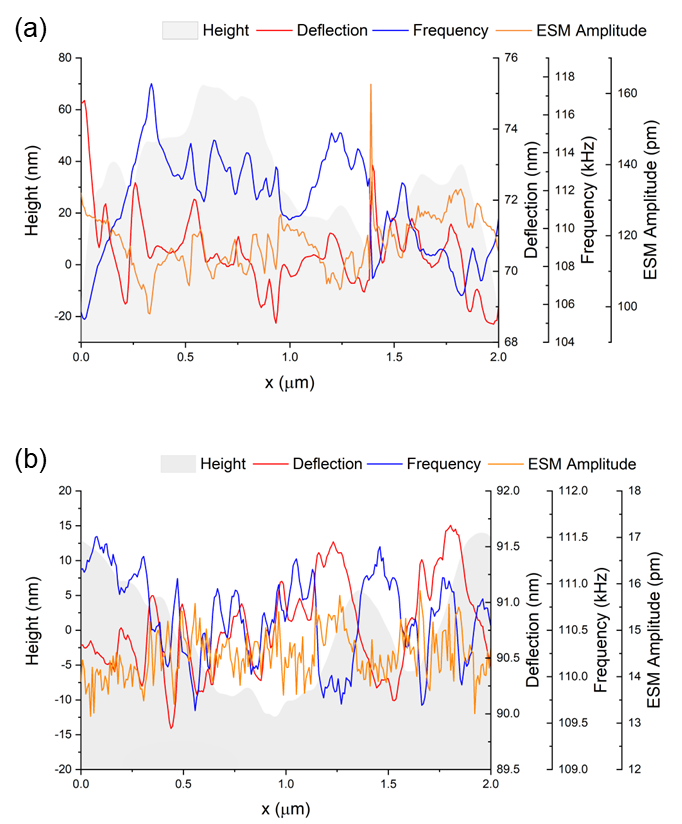


**Figure S12.** Line profiles of the AFM signals, including height, deflection, contact resonance frequency, and ESM amplitude, measured on NZTO solid electrolyte: (a) mechanically polished using sandpaper and (b) after CCP treatment.

1. Comparison of the normalized expected resonance frequency with the normalized experimentally measured frequency on NZTO solid electrolyte under sandpaper polishing and after CCP treatment


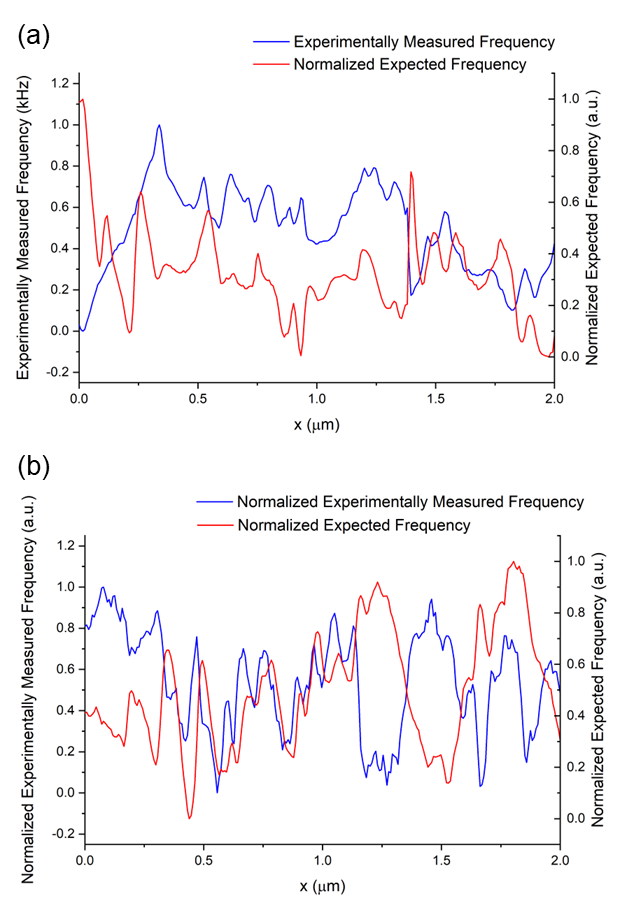


**Figure S13.** Comparison of the normalized expected resonance frequency with the normalized experimentally measured frequency on NZTO solid electrolyte: (a) mechanically polished using sandpaper and (b) after CCP treatment.
